# An Objective Pronator Drift Test Application (iPronator) Using Handheld Device

**DOI:** 10.1371/journal.pone.0041544

**Published:** 2012-07-24

**Authors:** Soojeong Shin, Eunjeong Park, Dong Hyun Lee, Ki-Jeong Lee, Ji Hoe Heo, Hyo Suk Nam

**Affiliations:** 1 Department of Neurology, Yonsei University College of Medicine, Seodaemoon-gu, Seoul, Korea; 2 Department of Computer Science and Engineering, Ewha Womans University, Seodaemoon-gu, Seoul, Korea; University of South Florida, United States of America

## Abstract

**Background:**

The pronator drift test is widely used to detect mild arm weakness. We developed an application that runs on a handheld device to objectify the pronator drift test and investigated its feasibility in stroke patients.

**Methods:**

The iPronator application, which uses the built-in accelerometer in handheld devices, was developed. We enrolled acute ischemic stroke patients (n = 10) with mild arm weakness and healthy controls (n = 10) to validate the iPronator. In addition to conventional neurological examinations, the degree of average, maximum, and oscillation in drift and pronation were measured and compared using the iPronator. Follow-up tests using the iPronator were also conducted in the patient group one week later.

**Results:**

There was a strong correlation between the average degree of pronation and drift measured by the iPronator (r = 0.741, p<0.001). The degrees of average and maximum in pronation were greater in the patient group than in the control group [in average, 28.9°, interquartile range (IQR) 18.7–40.3 vs. 3.8° (IQR 0.3–7.5), p<0.001], in maximum, 33.0° (IQR 24.0–52.1) vs. 6.2° (IQR 1.4–9.4), p<0.001]. The degree of oscillation in pronation was not different between the groups (p = 0.166). In drift, the degrees of average, maximum, and oscillation were greater in the patient group. In stroke patients, a follow-up study at one week revealed improvements in the degrees of pronation and drift compared with baseline parameters.

**Conclusions:**

The iPronator can reliably detect mild arm weakness of stroke patients and was also useful in detecting functional recovery for one week in patients with acute stroke.

## Introduction

Several examination methods have been developed to uncover mild motor weakness. The pronator drift test is widely used to detect mild arm weakness and to lateralize lesions. The pronator drift test is simple and easy, can be quickly performed by the patient’s bedside, and does not require additional equipment. However, regardless of its usefulness, the sensitivity of the pronator drift test is fairly low [1], [2]. The lack of objective parameters and reliance on the subjective decisions of the examiner limit the usefulness of the pronator drift test.

Accelerometers can be used to objectively measure real-time acceleration of motion [3]. Several studies have demonstrated that accelerometers are a reliable tool for quantifying physical activity and walking speed after stroke [3], [4]. The accelerometer is now a standard feature in most handheld devices, including smart phones and entertainment devices. Handheld devices are increasingly being used in the medical field for the diagnosis and treatment of patients and the training and education of medical personnel [5], [6].

We developed an objective pronator drift test application that runs on handheld devices and determined its feasibility and usefulness in patients with acute ischemic stroke.

## Methods

### Development of a Handheld Device Application

We developed an objective pronator drift test handheld device application, named the iPronator (http://itunes.apple.com/us/app/ipronator/id471884445?mt=8), using the iPhone software development toolkit (SDK 3.0, Apple Inc., Cupertino, CA, USA). The iPronator can measure the degrees of drift and pronation in real-time using the built-in accelerometer in the iPod touch. The iPod touch has a tri-axial accelerometer that measures acceleration in all three spatial dimensions; the x-axis (the short side), the y-axis (the long side), and the z-axis (a line perpendicular to the iPod touch display panel and through its center) [7]. In the iPronator, a change in the x-axis corresponds to pronation while a change in the y-axis reflects drift. Values are given in terms of the force of gravity. The arc sine function (asin) is used to convert the force of gravity data into radians. The radians are then converted into degrees using the following equation: degrees  =  radians ×180/pi. The accelerometer measures the changes in drift and pronation at 0.5 seconds intervals.

**Figure 1:**
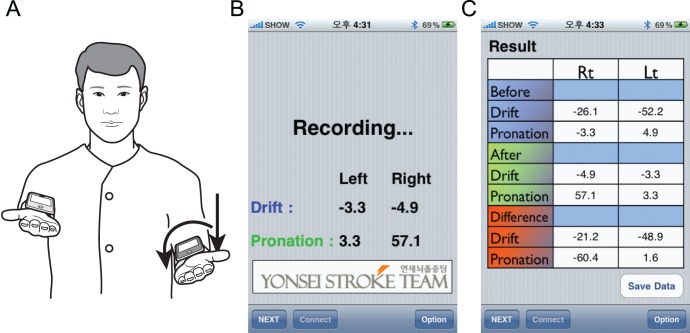
The iPronator is an application that runs on handheld devices. Two iPod touch devices were placed on each of the subject’s forearms and held firmly in place with Velcro above the wrists. In patients with mild arm weakness, drift (arrow) and pronation (curved arrow) were observed (A). The device displayed the real-time degree of drift and pronation in response to changes in the position of each arm (B). At the end of the exam, summary data were displayed and the raw data were saved on the handheld device (C).

A Bluetooth connection is used to transfer the data from a handheld device on each arm. One of the devices displays the real-time degrees of drift and pronation in response to a position change of the arm, while the other device displays the elapsed time. At the end of the exam, summary data are displayed and saved as raw data (Figure 1). In this study, the iPronator application was installed in an iPod Touch device. Because the iPod Touch has the same functions and display as the iPhone, the iPronator can also be installed and used in iPhones (Apple Inc., Cupertino, CA, USA).

### Study Subjects

Patients with acute ischemic stroke confirmed by diffusion-weighted MRI within 7 days from symptom onset were prospectively recruited. To be enrolled in this study, the patients were required to have mild arm weakness of the affected arm, confirmed by conventional pronator drift test. Conventional pronator drift test was performed by asking the patient to hold both forearms in supination, fully extending their elbows with a 90° extension forward at the shoulder joints, with eyes closed [2]. A positive pronator drift test was defined when the patient’s affected arm pronated or drifted downwards within 20 seconds. Patients who had substantial weakness of the affected arm, defined as a National Institute of Health Stroke Scale (NIHSS) score >3 or Medical Research Council (MRC) grade <II, were excluded. Patients who were not able to sit and those with bilateral arm weakness or preexisting chronic arm weakness were excluded. Patients who had a condition that could interfere with the pronator drift test results such as aphasia, neglect, peripheral neuropathy, myopathy, joint deformity, arthritis, or radiculopathy were also excluded. Healthy volunteers with no history of neurological disease and no weakness were enrolled as controls. This study was approved by the Severance Hospital Institutional Review Board and written informed consents were obtained from all patients and volunteers.

**Table 1:** Neurological examinations in the patient group.

	Sex/age	Affected side	NIHSS total	NIHSS arm	MRC proximal	MRC distal	Forearm rolling test	Finger rolling test
1	M/69	Lt	6	1	IV	IV	AbNL	AbNL
2	M/78	Lt	7	1	IV	IV	AbNL	AbNL
3	M/56	Lt	3	1	IV+	V	NL	AbNL
4	F/67	Rt	4	1	IV+	IV+	AbNL	AbNL
5	M/55	Lt	4	1	IV+	IV	AbNL	AbNL
6	F/71	Lt	1	0	V	V	NL	AbNL
7	M/79	Rt	5	2	III	II	AbNL	AbNL
8	F/83	Rt	4	1	IV+	IV+	AbNL	AbNL
9	F/71	Rt	3	1	IV	III	AbNL	AbNL
10	M/62	Lt	6	3	II	II	AbNL	AbNL

NIHSS = National Institutes of Health Stroke Scale scores; MRC = Medical Research Council grade; AbNL = abnormal, NL = normal.

**Figure 2:**
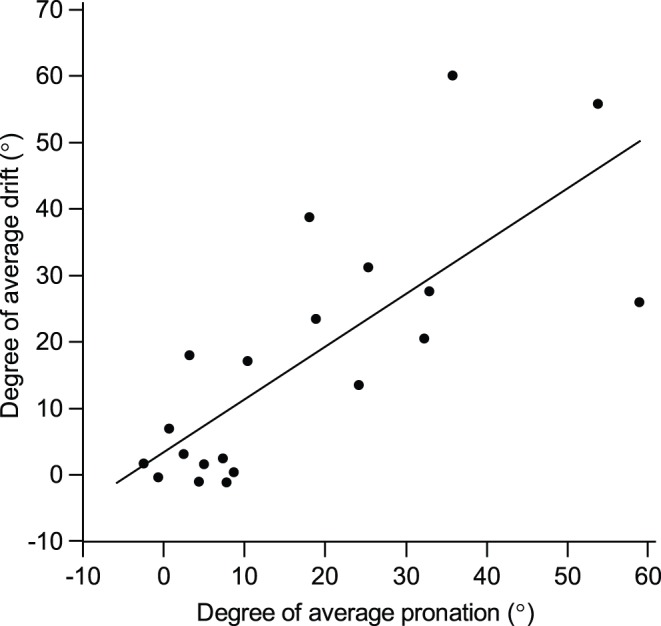
Correlation between degrees of pronation and drift. A strong correlation between the average degree of pronation and the average degree of drift is shown (r = 0.741, P<0.001).

**Figure 3:**
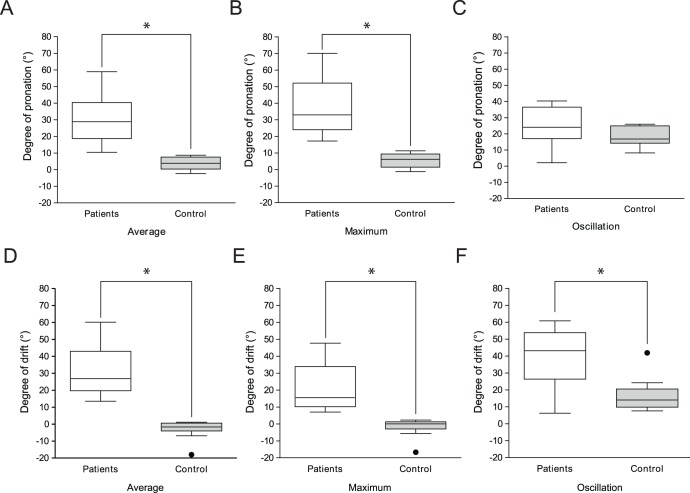
Comparison of the degrees of pronation and drift between patients and controls. The parameters of average (A), maximum (B) in pronation and the parameters of average (D), maximum (E), and oscillation (F) of drift were significantly greater in patients than in healthy controls whereas, the degree of oscillation in pronation was not different (C).

### Study Protocol

In addition to the conventional pronator drift test, the forearm rolling test and finger rolling test were administered to all subjects, and the NIHSS scores and MRC grades of all subjects were assessed by a neurologist (SS). In the forearm rolling test, each forearm was rapidly rotated around the other for 5 seconds, forwards and backwards. An abnormal response was defined when one forearm orbited around the other. In the finger rolling test, each index finger rotated around the other for 5 seconds, forwards and backwards. An abnormal response was defined when one finger orbited around the other [1].

Objective pronator drift test using the iPronator application was performed in each subject. The patients attempted the pre-test 3 times. If the patient could not perform the iPronator task, the patient was excluded. After the pre-test, the trial was conducted once for each patient. Two iPod Touch devices were placed on each of a subject’s forearms, and was held firmly in place with Velcro above the wrists. After attaching the devices, the subject raised his/her arms in the same manner as used for the conventional pronator drift test (Figure 1). A Bluetooth connection was established between the devices on each arm by touching the connection buttons on each device, and recording was initiated by touching the display panel. The examination was performed for 20 seconds with the patient’s eyes closed. Drift and pronation data were collected by the iPronator in real-time and the raw data were sent to a personal computer via e-mail. One of the investigators (HSN), blind to the group designation of the patients, reviewed the raw data. Only data recorded in the last 10 seconds were analyzed because the study patients needed to adjust the device weight and an initial dip was commonly observed in patients with upper extremity weakness.

We enrolled acute ischemic stroke patients (n = 10) with mild arm weakness and healthy controls (n = 10) to validate the iPronator. Along with the conventional neurological examinations, average, maximum, and oscillation changes in drift and pronation of the subjects were measured and compared using the iPronator. Follow-up tests using the iPronator were also conducted in the patient group one week later. An additional validation experiment was conducted in a different patient group (n = 10).

**Table 2:** Differences in degree of pronation and drift between patients and controls at baseline.

	Patients (n = 10)	Controls (n = 10)	p-value
Degree of pronation			
Average (°)	28.9 (18.7–40.3)	3.8 (0.3–7.5)	<0.001
Maximum (°)	33.0 (24.0–52.1)	6.2 (1.4–9.4)	<0.001
Oscillation (°)	24.0 (17.1–36.5)	16.8 (14.2–24.9)	0.166
Degree of drift			
Average (°)	26.8 (19.7–43.0)	−1.7 (−4.0–0.5)	<0.001
Maximum (°)	15.5 (10.2–33.9)	0.1 (−3.0–1.3)	<0.001
Oscillation (°)	43.2 (26.4–53.8)	14.0 (9.8–20.5)	0.007

Values are median (25 percentile–75 percentile).

### Statistical Analysis

SPSS software 18.0 for Windows (SPSS Inc., Chicago, IL, USA) and Graphad Prism version 5 (Graphad Software Inc., CA, USA) was used for statistical analyses. Because all parameters of pronation and drift were not normally distributed by the Kolmogorov-Smirnov test, we reported descriptive statistics as the median and interquartile range (IQR) and compared them using the non-parametric test of the Mann-Whitney U test and the Wilcoxon signed-rank test. Bivariate correlation analysis between paramerers was performed using the Spearman test. A *P* value less than 0.05 was considered significant.

**Table 3:** Differences in degree of pronation and drift between two arms.

	Affected arm	Unaffected arm	p-value
Degree of pronation			
Average (°)	28.9 (18.7–40.3)	0.5 (−1.2–2.1)	<0.001
Maximum (°)	33.0 (24.0–52.1)	3.5 (0.8–7.5)	<0.001
Oscillation (°)	24.0 (17.1–36.5)	22.2 (19.5–31.7)	0.940
Degree of drift			
Average (°)	26.8 (19.7–43.0)	−2.1 (−3.6–1.8)	<0.001
Maximum (°)	15.5 (10.2–33.9)	−4.9 (−7.15–−1.0)	<0.001
Oscillation (°)	43.2 (26.4–53.8)	15.1 (14.5–19.8)	0.007

Values are median (25 percentile–75 percentile).

**Figure 4:**
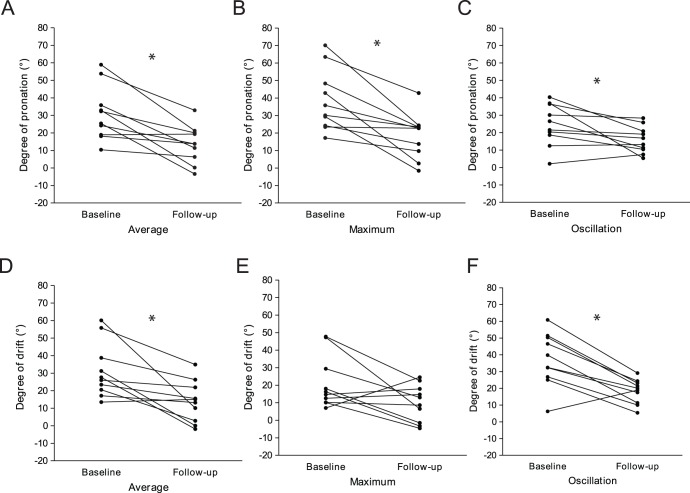
Improvements in the degree of pronation and drift during follow-up in the patient group. The degrees of average (A), maximum (B), and oscillation (C) of pronation were significantly improved from the baseline value. However, the degrees of average (D) and oscillation (F) in drift were significantly improved, the degree of maximum (E) drift was not different compared with baseline.

**Table 4:** Improvements in the degree of pronation and drift during follow-up in the patient group.

	Baseline (n = 10)	Follow-up (n = 10)	p-value
Degree of pronation			
Average (°)	28.9 (18.7–40.3)	13.8 (4.8–20.3)	0.004
Maximum (°)	33.0 (24.0–52.1)	22.7 (7.9–23.6)	0.002
Oscillation (°)	24.0 (17.1–36.5)	15.0 (9.7–22.1)	0.027
Degree of drift			
Average (°)	26.8 (19.7–43.0)	14.1 (2.1–23.0)	0.004
Maximum (°)	15.5 (10.2–33.9)	10.8 (−2.0–19.1)	0.106
Oscillation (°)	43.2 (26.4–53.8)	20.8 (11.0–25.5)	0.006

Values are median (25 percentile–75 percentile).

## Results

### Patients Versus Controls

A total of 10 patients (mean age 69.1±9.4 years, 6 of men) and 10 controls (mean age 40.1±10.0, 2 of men) were enrolled. All stroke patients had pronator drift in conventional pronator drift test measurements (4 on the right arm, 6 on the left arm). The total median NIHSS score in patient group was 4.0 (IQR 3.0–6.0), and the median NIHSS score of the affected arm was 1 (IQR 1–1.25). The forearm rolling test was positive in 8 out of 10 patients and the finger rolling test was positive in all patients (Table 1). Measurements from the iPronator demonstrated a strong correlation between the average degree of pronation and that of drift (r = 0.741, P<0.001) (Figure 2). Neither the NIHSS score nor the MRC grade was correlated with the degree of pronation or drift (data not shown). Moreover, 1 out of 10 patients showed normal muscle strength in the arm as measured by the NIHSS score or MRC grade. Baseline degrees of pronation and drift of the patient group were greater than those of the control group. The average degree of pronation in the patient group was 28.9° (IQR 18.7–40.3), which was greater than the control group [3.8° (IQR 0.3–7.5), P<0.001] (Figure 3A). The maximum pronation was also greater in the patient group [33.0° (IQR 24.0–52.1) vs. 6.2° (IQR 1.4–9.4), p<0.001] (Figure 3B) whereas, the oscillation of pronation was not different between the groups (Figure 3C). In regards to drift, all parameters were significantly greater in the patient group than the control group. The average degree of drift was greater in the patient group [26.8° (IQR 19.7–43.0)] than the control group [−1.7° (IQR −4.0–0.5)] (P<0.001) (Figure 3D). Both maximum (P<0.001) and oscillation of drift (P = 0.007) were also greater in the patient group than the control group (Figure 3E and F) (Table 2).

### Comparison between Affected and Unaffected Arm in Stroke Patients

In the patient group, the affected arms of patients showed greater pronation in the average degree (P<0.001) and the maximum degree (P<0.001) than the unaffected arms. In contrast, the oscillation degree of pronation was not different between the affected and unaffected arms (P = 0.940). The changes of all three parameters in drift of affected arms were greater than those of unaffected arms (the average degree of drift, P<0.001, the maximum degree of drift, P<0.001, and the oscillation degree of drift, P = 0.007) (Table 3).

### Improvements in the Degrees of Pronation and Drift during Follow-up in the Patient Group

A follow-up study for the same patients was conducted one week later. Slight neurological improvements in the affected arm measured by NIHSS scores were detected [from 1 (IQR 1–1.25) to 1 (IQR 1–1), P = 0.048]. Follow-up tests using the iPronator demonstrated the improvements of quantitative data. Comparing with baseline parameters, both parameters for pronation and drift were improved. The degrees of average (P = 0.004), maximum (P = 0.002), and oscillation (P = 0.027) of pronation were improved significantly at follow-up (Figure 4A to C). The degrees of average (P = 0.004) and oscillation (P = 0.006) of drift also improved significantly. However, the degree of maximum drift (P = 0.106) was not different between baseline and follow-up (Figure 4D to F) (Table 4).

### External Validation of the iPronator

External validation of the iPronator was conducted in the different patient group (n = 10). The characteristics of patients were not different from the first experiment except the patients with milder arm weakness (MRC grade >III) were enrolled (Table S1). All parameters of pronation and drift measured by the iPronator showed greater degree of changes in the patient group compared with the control group (Table S2).

## Discussion

This study demonstrated that the iPronator application was useful and feasible to objectify the pronator drift test. The parameters (average and maximum) of pronation and the parameters (average, maximum and oscillation) of drift were significantly different between the patients and healthy controls. In the stroke patients, the iPronator can detect improvements in the degrees of pronation and drift comparing with baseline values in follow-up study at one week.

The NIHSS score and MRC grade are commonly used to quantify motor weakness, but as we showed in this study, one of our patients had a normal NIHSS score and normal MRC grade despite a positive pronator drift test result, reflecting that these tools are not sufficient enough to measure the varying degree of weakness. Moreover, we found no correlation between the NIHSS score or MRC grade and objective parameters measured by the iPronator. Several other tests have been developed to detect mild upper extremity weakness such as the forearm rolling test, finger rolling test, rapid alternating movements of hands, rapid finger movements (tapping thumb to fingers or all fingers), fist opening/closing, and the shoulder shrug test. These tests are qualitative in nature, with positive or negative results; they tend to have high specificity but low sensitivity (11% to 33%) [1], [2]. Combinations of these tests only increase the detection rate to 50% in patients with focal brain lesions [1]. Our study also showed that despite positive pronator drift test results, two patients had negative forearm rolling test. Although these tests are clinically useful, the lack of objective parameters is one of their major limitations [8]. Taken together, conventional motor tests and scales fail to detect all cases of mild arm weakness.

The iPronator application, by exploiting the built-in accelerometer of handheld devices, can be used to determine the degrees of drift and pronation in real-time. The correlation analysis between the degrees of pronation and drift determined by the iPronator was high. Although the average and maximum pronation and drift values showed significant differences between the affected arm and unaffected arm in the stroke patients, the oscillation of pronation was not significantly different between the affected and unaffected arms. This lack of difference in oscillation may be due to the counter-movements of the arms; counter-movements of one limb make the other limb move in the opposite direction [9], thus the affected arm may cause the unaffected arm to balance the posture.

Besides the ability of iPronator in detecting mild arm weakness, the iPronator can be applied to monitor the improvement or progression of motor weakness. A follow-up study conducted at one week later showed that the degrees of average, maximum, and oscillation of pronation improved significantly at follow-up. The degrees of average and oscillation of drift also improved significantly. However, the degree of maximum drift was not different between baseline and follow-up. Taken together, the degrees of average pronation and drift measured by the iPronator might be the most useful parameter in both detection and follow-up of mild arm weakness in stroke patients.

Handheld devices are becoming more widely used to diagnose and treat various diseases [10]–[12]. Current handheld devices are particularly suited for medical purposes because of their rich multi-touch user interfaces, built-in accelerometers, location-sensing frameworks, fast processors, and easily available network connections [7]. Additional benefits of handheld devices include easy portability and accessibility. Physicians are becoming increasingly familiar with handheld devices [6], [13], and little effort is required to learn how to use applications that run on handheld devices.

This study has several limitations. First, the sensitivity or specificity of the iPronator could not be determined because the patients with abnormal pronator drift test were selectively enrolled. The ability of the iPronator to detect subtle arm weakness needs to be confirmed after including all patients with focal cerebral lesions. Second, the detection of pronator drift is just one part of the neurological examination. The iPronator cannot be used as a sole screening tool for evaluating stroke patients.

In this study, we demonstrated that the iPronator application was useful and feasible in the detection of mild arm weakness and to quantify the degree of weakness. Moreover, the iPronator was also useful in detecting functional recovery for one week in patients with acute stroke.

## Supporting Information

Table S1 Clinical characteristics and results of neurological examinations in the patients for external validation. The characteristics of patients were not different from the first experiment except the patients with milder arm weakness were enrolled in the external validation.
(DOC)

Table S2 Differences in degrees of pronation and drift between the patients for external validation and controls. All parameters of pronation and drift measured by the iPronator showed greater degree of changes in the patient group compared with the control group.
(DOC)
