# Supplementary material for: An Objective Pronator Drift Test Application (iPronator) Using Handheld Device
Source: PLoS One. 2012 Jul 24;7(7):e41544. doi: 10.1371/journal.pone.0041544 (PMC3404034; doi:10.1371/journal.pone.0041544)
Supplement: Table S1 — Clinical characteristics and results of neurological examinations in the patients for external validation. The characteristics of patients were not different from the first experiment except the patients with milder arm weakness were enrolled in the external validation. (DOC) [file pone.0041544.s001.doc]

**ONLINE SUPPLEMENT**

**Table S1 Clinical characteristics and results of neurological examinations in the patients for external validation.**

|  | Sex/age | Affected side | NIHSS total | NIHSS arm | MRC proximal | MRC distal | Forearm rolling test | Finger rolling test |
| --- | --- | --- | --- | --- | --- | --- | --- | --- |
| 1 | M/53 | Rt | 3 | 1 | IV | IV+ | AbNL | AbNL |
| 2 | M/79 | Rt | 5 | 1 | IV+ | IV+ | AbNL | AbNL |
| 3 | M/52 | Lt | 6 | 1 | IV+ | IV+ | AbNL | AbNL |
| 4 | M/76 | Rt | 12 | 2 | III+ | IV- | AbNL | AbNL |
| 5 | F/67 | Rt | 2 | 1 | IV | V | NL | NL |
| 6 | M/59 | Rt | 9 | 0 | V | V | AbNL | AbNL |
| 7 | F/76 | Lt | 3 | 1 | IV+ | V | NL | AbNL |
| 8 | M/73 | Rt | 2 | 1 | IV | IV+ | AbNL | AbNL |
| 9 | F/77 | Lt | 4 | 1 | IV | IV | AbNL | AbNL |
| 10 | M/45 | Lt | 1 | 0 | V | V | AbNL | AbNL |

NIHSS = National Institutes of Health Stroke Scale score; MRC = Medical Research Council grade; Rt = right, Lt = left; AbNL = abnormal, NL = normal.
