# Supplementary material for: An Objective Pronator Drift Test Application (iPronator) Using Handheld Device
Source: PLoS One. 2012 Jul 24;7(7):e41544. doi: 10.1371/journal.pone.0041544 (PMC3404034; doi:10.1371/journal.pone.0041544)
Supplement: Table S2 — Differences in degrees of pronation and drift between the patients for external validation and controls. All parameters of pronation and drift measured by the iPronator showed greater degree of changes in the patient group compared with the control group. (DOC) [file pone.0041544.s002.doc]

**ONLINE SUPPLEMENT**

**Table S2 Differences in degrees of pronation and drift between the patients for external validation and controls.**

|  | Patients (n = 10) | Controls (n = 10) | p-value |
| --- | --- | --- | --- |
| Degree of pronation | | | |
| Average (°) | 23.1 (14.0 - 35.4) | 3.8 (0.3 - 7.5) | 0.006 |
| Maximum (°) | 28.8 (16.1 - 39.2) | 6.2 (1.4 - 9.4) | 0.012 |
| Oscillation (°) | 23.9 (20.2 - 33.5) | 16.8 (14.2 - 24.9) | 0.017 |
| Degree of drift | | | |
| Average (°) | 15.9 (9.3 - 29.6) | –1.7 (–4.0 - 0.5) | 0.003 |
| Maximum (°) | 25.5 (13.3 - 45.9) | 0.1 (–3.0 - 1.3) | 0.014 |
| Oscillation (°) | 35.3 (23.9 - 56.8) | 14.0 (9.8 - 20.5) | 0.026 |

Values are median (25 percentile – 75 percentile).
